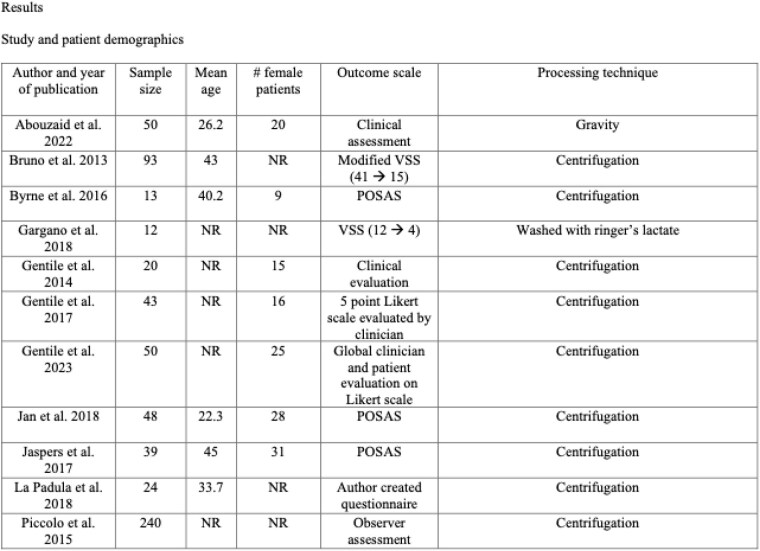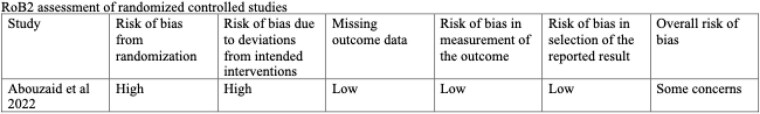# 681 A Systematic Review of Fat Grafting in the Management of Burn Scars

**DOI:** 10.1093/jbcr/iraf019.310

**Published:** 2025-04-01

**Authors:** Xi Ming Zhu, Omar El Sewify, Cory Tremblay, Éolie Delisle, Shahriar Shahrokhi

**Affiliations:** McMaster University; McGill University; Northern Ontario School of Medicine University; University of Montreal; McMaster University

## Abstract

**Introduction:**

Secondary management of thermal injuries remains a challenging and relevant topic for plastic surgeons. Burn scars are associated with both functional and aesthetic issues for patients. While various treatments exist, there is no gold standard of treatment. Fat grafting has been used in multiplicities in reconstruction, and studies have demonstrated improvement in skin texture and contour following infiltration. This systematic review aims to examine all available evidence on outcomes following fat grafting for management of burn scars.

**Methods:**

A search of Medline, EMBASE, and Cochrane library database was conducted from their inception until June of 2023. Published articles examining outcomes of fat grafting for thermal injury scars were identified, screened, and extracted as per PRISMA guidelines.

**Results:**

A total of 10457 articles were screened, yielding 14 eligible studies for data extraction accounting for 885 patients. All studies found improvement post-operatively. 9 out of 14 studies used a subjective clinical assessment, one study did not report pre-treatment measurements, the other 8 all found improved outcome based on clinician assessment. One study reported VSS and another reported modified VSS. 3 studies utilized POSAS, the mean difference was an improvement of 7.28 (MCID < 1).

**Conclusions:**

Further studies, particularly prospective in nature, with standardized outcome measurements are needed to substantiate clinical improvement observed. The authors recommend utilizing either the POSAS or VSS for future studies investigating burn scar treatments.

**Applicability of Research to Practice:**

This study investigates all concurrent and relevant studies looking at utilizing fat grafting in the management of burn scars. Evidence present is overall supportive of its efficacy. However, meaningful analysis was precluded due to inconsistent outcome reporting. We recommend all studies going forward on this topic to use established tools or PROMs such as the POSAS or VSS.

**Funding for the Study:**

N/A